# Berberine-Coated Biomimetic Composite Microspheres for Simultaneously Hemostatic and Antibacterial Performance

**DOI:** 10.3390/polym13030360

**Published:** 2021-01-22

**Authors:** Xiaojian Zhang, Kaili Dai, Chenyu Liu, Haofeng Hu, Fulin Luo, Qifan Qi, Lei Wang, Fei Ye, Jia Jin, Jie Tang, Fan Yang

**Affiliations:** 1 Shanghai Engineering Research Center of Molecular Therapeutics and New Drug Development, East China Normal University, Shanghai 200062, China; 51184300155@stu.ecnu.edu.cn (X.Z.); 15988810463@163.com (K.D.); 51194300147@stu.ecnu.edu.cn (C.L.); 2 College of Life Sciences and Medicine, Zhejiang Sci-Tech University, Hangzhou 310018, China; 201920201014@mails.zstu.edu.cn (H.H.); 202020801049@mails.zstu.edu.cn (F.L.); 2018339901070@mails.zstu.edu.cn (Q.Q.); leiwang1986@hotmail.com (L.W.); yefei@zstu.edu.cn (F.Y.); 3 Zhejiang Provincial Key Laboratory of Silkworm Bioreactor and Biomedicine, Hangzhou 310018, China

**Keywords:** alginate, cellulose, gelatin, biomimetic microsphere, hemostasis, antibacterial

## Abstract

Biomimetic microspheres containing alginate/carboxymethylcellulose/gelatin and coated with 0%, 1%, 3%, and 6% berberine (BACG, BACG-1B, BACG-3B, BACG-6B) were prepared by the oil-in-water emulsion method combined with spray drying. Through a series of physicochemical parameters and determination of hemostatic properties in vitro and in vivo, the results indicated that BACG and BACG-Bs were effective in inducing platelet adhesion/aggregation and promoting the hemostatic potential due to their biomimetic structure and rough surface. In addition, BACG-6B with high berberine proportion presented better hemostatic performance compared with the commercial hemostatic agent compound microporous polysaccharide hemostatic powder (CMPHP). BACG-6B also showed strong antibacterial activity in the in vitro test. The hemolysis test and cytotoxicity evaluation further revealed that the novel composite biomaterials have good hemocompatibility and biocompatibility. Thus, BACG-6B provides a new strategy for developing a due-functional (hemostat/antibacterial) biomedical material, which may have broad and promising applications in the future.

## 1. Introduction

Traumatic massive bleeding has been the leading cause of death in surgery and war [1,2,3,4,5,6]. On the one hand, excessive blood loss can lead to lower blood pressure, difficulty breathing, even coma, and other life crisis symptoms. On the other hand, the secondary infection that occurs after wound hemorrhage induces tetanus and septicemia easily. In severe cases, it can lead to septic shock, a life-threatening condition that has attracted a lot of attention in the clinic [7]. Hence, developing hemostatic agents with antibacterial effect to control bleeding and prevent wound infection is highly desirable and urgent.

At present, polysaccharide and polypeptide hemostatic agents account for a large proportion in the market. Alginate, a natural product with good hemostasis and cytocompatibility extracted from algae, has been widely used in drug delivery, hemostasis, and wound healing [8,9,10,11,12]. Sodium alginate (SA) is often used in various composite hemostatic agents since Na^+^ is easily ion-exchanged with Ca^2+^, forming a cross-network structure [9]. The molecules contain a large number of carboxyl groups as a highly negatively charged structure showing high biocompatibility and degradability, which are reflected as anions in an aqueous solution [13]. Cellulose, the most abundant natural polymer with wide sources, is a commercially used biomaterial [14,15,16]. Sodium carboxymethyl cellulose (SCC), a carboxymethyl derivative of cellulose, is the most industrial-scale product, playing an important role in food and drug preparations due to its low cost, renewability, biocompatibility, and hydrophilicity. SCC has excellent water-absorbing ability as its numerous hydroxyl and carboxylic groups bind with water, which further form a viscous gel and promote platelet aggregation at the bleeding site, thus achieving the purpose of hemostasis. Gelatin, a polypeptide of collagen hydrolytic and thermal degradation, is widely sourced and used in various medical applications [17]. Because of its excellent functions of hydrophilicity, biodegradation, and biocompatibility [18], gelatin has been employed in wound healing [19] and skeleton support as a scaffold material [20,21]. The liquid condenses in a gel void, which is conducive to maintaining the stable shape of the polymer and withstanding a large load. Therefore, gelatin can help polymers to activate plasma proteins with no damage to blood cells [17]. However, due to its slow hemostasis speed and poor viscoelasticity, it is often combined with polysaccharides to achieve hemostasis. 

It is difficult for a single biomaterial to meet all needs of hemostasis, and combined use of multiple materials will help eliminate the disadvantages, improve physicochemical properties, and enhance hemostatic performance. In recent years, a series of new composite polysaccharide hemostatic materials have been recognized and developed [22,23]. Cheng et al. successfully prepared a biodegradable, non-inflammatory oxide nanocrystalline cellulose/SA composite hemostatic material that achieved rapid hemostasis by erythrocyte adsorption and platelet aggregation [24]. Zhu et al. showed that ZnO/bioactive glass nanoparticles in alginate/chitosan composite hydrogels significantly accelerate wound closure because their Si^4+^ and Ca^2+^ ions stimulate fibroblasts to secrete various growth factors for angiogenesis and wound closure [25]. Leppiniemi et al. prepared 3D-printable SCC/SA composite hydrogels suggesting potential applications as wound dressings [26]. Although antibacterial dressings for wound healing are widely available [27], there are still few antibacterial applications of hemostatic powder that help prevent wound infection. Therefore, it is urgent to develop a safe, efficient, biocompatible, and antibacterial hemostatic powder [28]. Berberine, the main active component of *Coptis chinensis* Franch., is considered to have good natural antibacterial activity, especially against Gram-positive bacteria [29,30]. The antibacterial mechanism of berberine includes interfering with DNA replication, RNA transcription, protein synthesis, etc. [31,32]. The analysis of the chemical structure shows that the quaternary ammonium structure (aromatic ring of positively charged N atoms) is necessary for berberine’s antibacterial activity. Through an in-depth understanding of berberine, it is speculated that berberine may enhance hemostasis and anti-infection effects when it is used in combination with polysaccharides. In previous research, we found a series of SCC-B microspheres containing berberine that simultaneously facilitate hemostatic and antibacterial performance [33]. These results confirmed our speculation and further encouraged us to develop novel composite microspheres coated with berberine.

**Scheme 1:**
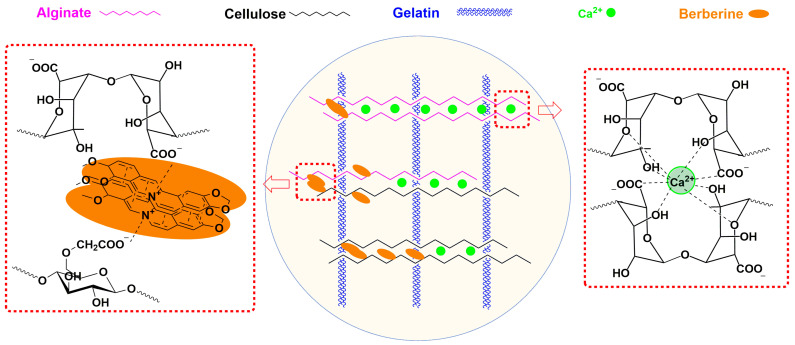
Schematic overview of BACG-B scaffolds and its possible cross-linking mechanism.

In this work, SA, SCC, and gelatin were used as raw materials to synthesize biomimetic alginate/carboxymethylcellulose/gelatin (BACG) through the emulsification and cross-linking process. SA has strong swelling capacity, SCC can activate erythrocytes and platelets, and gelatin can support a stable and biocompatible skeleton, promoting platelet aggregation [34,35]. Combining these three materials helps in sharing their advantages and avoiding their disadvantages. On this basis, berberine was further added to prepare novel due-functional (antibacterial/hemostatic) biomaterials. The addition of berberine not only gives hemostatic materials the strong ability to resist infection, but also helps in it being employed as a cross-linking regulator to replace the Ca^2+^ inserted into the original cross-linking system (Scheme 1). The composite structure of biomimetic microspheres, their physical properties (such as swelling and degradation), and their functional studies (including hemostatic performance in vitro/in vivo, interaction of platelets, antibacterial activity, and biocompatibility) are comprehensively investigated.

## 2. Materials and Methods

### 2.1. Materials

SCC was obtained from Beijing Solebao Technology Co., Ltd. (Beijing, China). SA was purchased from Qingdao Huanghai Biopharmaceutical Co., Ltd. (Qingdao, China). Gelatin (MW: 115,000–130,000) was bought from Sinopharm Chemical Reagent Co., Ltd. (Shanghai, China). Compound microporous polysaccharide hemostatic powder (CMPHP) was purchased from Shandong Success Pharmaceutical Technology Co., Ltd. (Jinan, China). Berberine chloride (MW: 371.81, 98%) was purchased from Shanghai Macklin Biochemical Co., Ltd. (Shanghai, China). Dimethylsulfoxide (DMSO), 3-(4,5-dimethylthiazol-2-yl)-2,5-diphenyltetrazolium bromide (MTT) and trypsin were purchased from Sigma (St. Louis, MO, USA). The myoblast cell line (L6) was obtained from the Shanghai Institute of Cell Biology, Chinese Academy of Sciences (Shanghai, China). Bacterial strains of *Staphylococcus aureus* (*S. aureus*) were obtained from the China Center for Type Culture Collection (CCTCC) (Wuhan, Hubei, China). All other reagents and solvents (analytical grade) were available from Sinopharm Chemical Reagent (Shanghai, China).

### 2.2. Preparation and Characterization

#### 2.2.1. Preparation

SA, SCC, and gelatin were mixed and dissolved in water (25 mg/mL) in the ratio of 60/40/1. The mixture was later emulsified with Tween-80 for 30 min. Then, the cross-linking agent (2% CaCl_2_ solution) was added dropwise and the mixture stirred for 60 min. After that, the sample was soaked and precipitated in anhydrous ethanol and washed with alcohol/petroleum ether. After washing, the microsphere was dissolved in 20% ethanol solution again and different amounts of berberine (0%, 1%, 3%, and 6% of the total weight of the microsphere) were added to prepare different hemostatic materials. Finally, a series of red blood cell (RBC)-mimetic microspheres (BACG, BACG-1B, BACG-3B, and BACG-6B) were obtained by spray drying.

#### 2.2.2. Surface Morphology Characterization by Scanning Electron Microscopy

Samples of BACG, BACG-1B, BACG-3B, and BACG-6B were directly mounted on a holder using double-sided conductive adhesive tapes after being sputter-coated with gold. Then, scanning electron microscopy (Hitachi S-4800, Hitachi Ltd., Tokyo, Japan) was employed to analyze the surface morphology of the samples.

#### 2.2.3. Fourier Transform Infrared Spectroscopy (FT-IR)

The characteristics of the different raw materials of BACG and various BACG-B were determined by the Fourier Transform Infrared Spectroscopy Nicolet spectrometer (Thermo Nicolet 5700; Thermo Fisher Scientific, Waltham, MA, USA) at room temperature. The FT-IR spectrum of each of the samples was mixed with KBr to laminate them for the tests in the wavelength range of 500–4000 cm^−1^ at a resolution of about 2 cm^−1^.

#### 2.2.4. Swelling

Firstly, the empty centrifuge tubes (*W*) and the centrifuge tubes with 10 mg composite materials (*W_i_*) were weighed. Phosphate-buffered saline (PBS; 2 mL) was added to the shaking tubes, which were placed in a water bath at 37 °C. After taking them out at different intervals, the tubes were centrifuged at 12,000 rpm for 2 min, and the supernatant was removed and weighed (*W_s_*). The same experiment was repeated three times in parallel. Finally, the swelling ratio (*SR*) was calculated according to Equation (1):

*SR* = (*W_s_* − *W_i_*)/(*W_i_* − *W*) × 100% (1)

#### 2.2.5. Degradation

The degradation of these materials was measured after they reached their maximal swelling weight. The experimental procedure was similar as the swelling assay. After the materials reaching the maximum swelling ratio, the degradation assay started by using 0.2% lysozyme solutions replaced PBS to simulate degradation environment in vivo. Finally, the degradation rate of all materials was measured as the degradation ratio (*DR*) until the total weight of the sample became stable. Three replicate experiments were performed, and the maximal swelling weight was set as 100%. The *DR* was calculated according to Equation (2):

*DR* = (*W_s_* − *W*)/(*W_ms_* − *W*) × 100% (2)

(*W_ms_*: the weight of tubes in maximal swelling after removing the supernatant)

### 2.3. Hemostatic Performance

All animal procedures were carried out with the Animal Care Committee’s approval, and the animals were cared for and studied in this research according to the normal regulations administered by the Animal Experimental Center of Hangzhou Normal University.

#### 2.3.1. Blood Collection

A normal and healthy New Zealand white rabbit (purchased from the Animal Experimental Center of Hangzhou Normal University, Hangzhou, China) was selected to collect whole blood in anticoagulant tubes. After centrifuging three times at 800 rpm for 8 min, the supernatant was collected to prepare platelet-rich plasma (PRP) [36]. 

#### 2.3.2. Whole Blood Coagulation In Vitro

Four prepared hemostatic materials and CMPHP (30 mg) were respectively placed into disposable plastic tubes with 500 μL of blood containing anticoagulant (3.8% sodium citrate/blood = 1/9). The tubes were oscillated on a vortex shaker for 5 s. The tubes were inverted every 100 s to observe the experimental phenomenon. If the blood was flowable, the tubes would reset until the blood completely coagulated or time was over 600 s. The time required for complete coagulation was recorded. Samples 1 to 5 were represented as CMPHP, BACG-1B, BACG-3B, BACG-6B, and blank control (BC, without any hemostatic materials), respectively. 

#### 2.3.3. Rat Tail Amputation Model

Sprague Dawley (SD) rats (♂, 0.2–0.25 kg, purchased from the Animal Experimental Center of Hangzhou Normal University, Hangzhou, China, project identification code is 2016C31017) were anesthetized by an intraperitoneal injection with 10% chloral hydrate (0.03 mL/kg). Half of the rats’ tails were cut off by surgical scissors. After that, pre-weighed tubes containing 100 mg samples (BACG, BACG-1B, BACG-3B, BACG-6B, and CMPHP) were placed to cover the wounds with minimal pressure by immersing the tails into the powdery samples. The clotting time (s) and blood loss (g) were recorded during the hemostatic process. A tube with CMPHP and an empty tube were used as the positive control group and the blank control group, respectively. A parallel group (*n* ≥ 6) was tested to obtain an average value.

#### 2.3.4. Platelet Aggregation

BACG, BACG-6B, and CMPHP (3.5 mg per sample) were mixed with 1 mL PRP. After incubating for 1 min, 3 min, and 5 min, 100 μL of the supernatant was collected from each tube. After dilution, the number of platelets was counted by an inverted fluorescence microscope. The number of platelets before aggregation (*BA*) was set as the blank control. A parallel group (*n* = 3) was tested to obtain an average value.

The aggregation ratio (*AGR*) of the platelets was calculated according to Equation (3):

*AGR* = (*BA* − *AA*)/*AA* × 100% (3)

(*BA*: before aggregation; *AA*: after aggregation)

#### 2.3.5. Platelet Adhesion

PRP was pipetted with a 5 mL syringe, and the microinjection pump was assembled according to the literature method [37]. The bottom of the syringe was covered with medical nylon cloth (500 mesh) and hemostatic materials (45 mg). The flow rate of the syringe pump was set to 0.5 mL/min, and the exudate was collected per 0.5 min. The number of platelets in the exudate was counted using an inverted fluorescence microscope. The platelet count before perfusion was set as the blank control.

The adhesion ratio (*ADR*) of the platelets was calculated according to Equation (4):

*ADR* = (*BP* − *AP*)/*BP* × 100% (4)

(*BP*: before perfusion; *AP*: after perfusion)

### 2.4. Antibacterial Activity

A nutrient agar containing *Staphylococcus aureus* (*S. aureus*) was prepared. The plate was divided into five parts on average, and small pieces of paper soaked in test samples (1 mg/mL ampicillin (Amp^+^) solution, 1 mg/mL berberine solution, 1 mg/mL and 10 mg/mL BACG-6B solution) were placed on the surface, respectively. A small piece of paper immersed in distilled water was placed in the center of the plate as a blank control (BC). The plate was incubated in 37 °C for 12 h. Finally, the bacteriostatic circle was observed and measured.

### 2.5. Biocompatibility

#### 2.5.1. Cytotoxicity Evaluation

The cytotoxicity of the hemostatic materials (BACG, BACG-1B, BACG-3B, BACG-6B, and CMPHP) was tested by MTT assay using L6 cells (mouse myoblast cell line). Three different concentrations of the experimental materials were set for testing: 1 mg/L, 10 mg/L, and 100 mg/L. Besides, the cytotoxicity of berberine was also assessed at the same three concentrations. Briefly, all materials were diluted into a suitable test concentration using PBS and then dissolved at 37 °C. The cells were cultured (90 μL/well) in 96-well microtiter plates and incubated at 37 °C in a humidified atmosphere of 5% CO_2_ for 24 h. Then, the lysate of the material or the drug (10 μL) was added to the 96-well plates for a further 72 h of incubation. After that, the cells were treated with MTT solution (5 mg/mL, 10 μL/well) for another 4 h at 37 °C. Finally, the absorbance was measured at 490 nm through an ultraviolet spectrophotometer (Shanghai Mapada Instruments Co., Ltd., Shanghai, China), which could indirectly reflect the number of living cells.

The cell viability (*CV*) was calculated according to Equation (5):

*CV* = (*A_s_* − *A_b_*)/(*A_c_* − *A_b_*) × 100% (5)

(*A_s_*: absorbance of the sample; *A_c_*: absorbance of the control group; *A_b_*: absorbance of the blank group)

#### 2.5.2. Hemolysis Test

The rabbit erythrocyte suspension (RES) obtained from the whole blood of the New Zealand white rabbit was stored with 5% citrate sodium (anticoagulants) for the hemocompatibility evaluations of the materials. To obtain the test RES (diluted with saline), the 2% red blood cells (RBCs) were resuspended in an isotonic saline solution. Test samples (10 mg) were separately immersed in a sterile, pyrogen-free normal saline solution (1 mL) for 24 h at 37 °C. Then, the extracts were prepared for further testing. In this experiment, normal saline (0.9% NaCl solution) as a negative control (NC) and distilled water as a positive control (PC) were prepared for hemolysis.

The extracts were mixed with RES according to Table S1 (Support Information), and the final concentration of each test group was 0.6 mg/mL. Then, the mixture was immediately incubated at 37 °C for 3 h to observe the hemolysis and coagulation reaction. Finally, the mixture was centrifuged at 1500 rpm for 10 min and the supernatant was collected to measure the absorbance values at 562 nm.

Eventually, the hemolysis ratio (*HR*) was calculated according to Equation (6):

*HR* = (*A_TS_* − *A_NC_*)/(*A_PC_* − *A_NC_*) × 100% (6)

(*A_TS_*: absorbance of the test sample; *A_NC_*: absorbance of the negative control; *A_PC_*: absorbance of the positive control)

## 3. Results

### 3.1. Characterization

#### 3.1.1. Scanning Electron Microscopy

**Figure 1:**
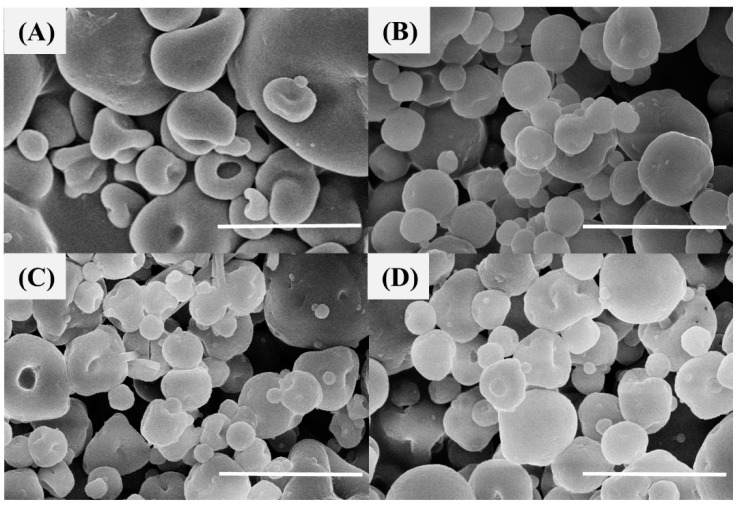
SEM images of microspheres. (**A**) biomimetic alginate/carboxymethylcellulose/gelatin (BACG); (**B**) BACG-1B; (**C**) BACG-3B; (**D**) BACG-6B (scale bar = 5 μm).

Inspired by red blood cells (RBCs), commonly known as an important member for activating blood coagulation [38], RBC-mimetic microspheres were constructed by the implementation of a biomass-derived polymer coated with berberine as an efficient medium for coagulation and antibacterial element loading. SEM was employed to clearly observe the sizes and structural features of these materials. As shown in Figure 1, we found that some materials were spheroidal in shape and some were biconcave discoid, shaped like RBCs, which exhibited a certain internal space elasticity to absorb liquid and reduce the volume expansion ratio. Most particles were 1–10 μm in diameter, similar in size to RBCs or platelets. In addition, microspheres with a rough surface help themselves adhere to the tissue, further promote the aggregation of RBCs and platelets, and form a blood coagulation network. In general, the biconcave hollow cavity with abundant liquid adsorption and catalytic conversion sites could confine active platelets outside and improve calcium ion transportation inside and outside.

#### 3.1.2. Fourier Transform Infrared Spectroscopy (FT-IR)

**Figure 2:**
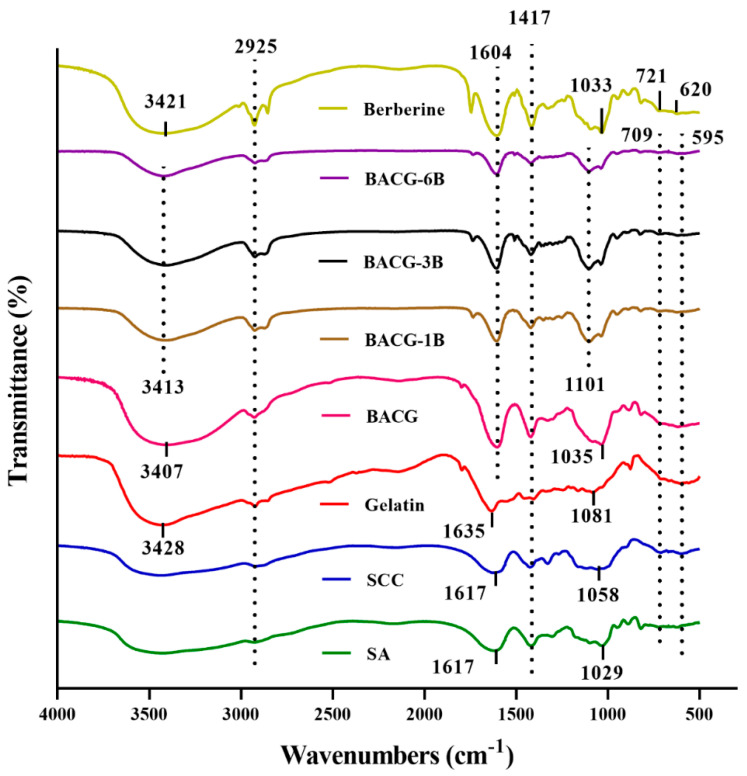
FT-IR spectra of BACG, BACG-1B, BACG-3B, BACG-6B and the raw materials (sodium alginate (SA), sodium carboxymethyl cellulose (SCC), gelatin, and berberine).

To demonstrate the characteristic functional groups of BACG and BACG-B, FT-IR analysis was carried out (Figure 2). Due to the abundant –OH groups on the surface of the materials, a broad overlapping band between 3000 cm*^−^*^1^ and 3750 cm*^−^*^1^ was observed in the spectra of SA, SCC, BACG, and BACG-B. In addition, the other intense composite band between 3000 cm*^−^*^1^ and 3700 cm*^−^*^1^ was attributed to the overlapping of O–H and N–H stretching vibrations (gelatin, SCC, BACG, and BACG-B). Moreover, the broad peaks in the BACG and BACG-B spectra confirmed the formation of a cross-linking network by intermolecular hydrogen bonding among SA, SCC, and gelatin. The absorption band at 2925 cm*^−^*^1^ was attributed to the C–H stretching vibration, and the absorption bands at 1417 cm*^−^*^1^ and 1320 cm*^−^*^1^ were attributed to the C–H symmetric and asymmetric bending vibrations, respectively. The peak at approximately 1600 cm*^−^*^1^ was attributed to the C=O stretching vibration, and the strong absorption peak at approximately 1030 cm*^−^*^1^ was attributed to the C–O–C stretching vibration (except gelatin). Furthermore, comparing the FT-IR spectra of BACG-B and BACG revealed that berberine was incorporated into the BACG to form BACG-B, attributing the broad absorption at approximately 843–499 cm*^−^*^1^ to the polynuclear aromatic C–H bending vibration and skeletal vibration of berberine. 

#### 3.1.3. Swelling

**Figure 3:**
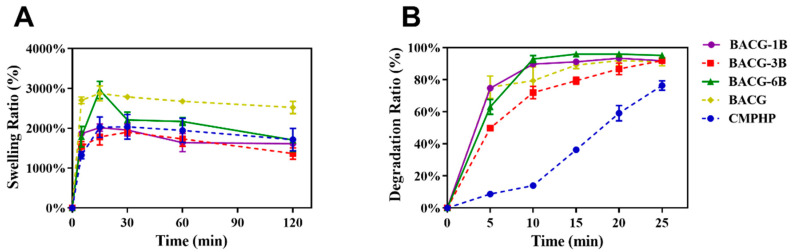
The swelling and degradation behaviors of BACG, BACG-1B, BACG-3B, BACG-6B, and CMPHP. (**A**) The swelling behaviors in phosphate-buffered saline (PBS); (**B**) the degradation behaviors in lysozyme solution. The data correspond to the mean ± SD (*n* = 3).

As reported previously, absorbing liquid to enrich blood cells and speed up the clotting process is a critical factor in a hemostatic agent [39]. Figure 3A shows the swelling peaks of all test materials were generally reached around 15 min. BACG and BACG-6B presented stronger swelling abilities (about 3000%) compared with BACG-1B, BACG-3B, and CMPHP in the initial 15 min, which was enough time for most hemostatic conditions. The reason why both BACG and BACG-6B presented the highest swelling ratio was that berberine acted as a cross-linking regulator (Scheme 1). Firstly, berberine could embed into the cross-linking system of BACG, replace the original Ca^2+^ cross-linking site, and further induce the cross-delinking of BACG, which resulted in a decline in the swelling ability. Then, the new cross-linking site formed by π-π interaction of intermolecular forces with the increase in the berberine content helped to reinforce the cross-linking system of microspheres and further enhanced the swelling ability. The above results indicated that BACG and BACG-6B had a strong water absorption ability, which benefited them as rapid hemostatic materials.

#### 3.1.4. Degradation

Biodegradability is a precondition for potential hemostats used in vivo. Hence, we tested the degradation behaviors in vitro after the swelling test. After swelling to the maximum water absorption, lysozyme was added to simulate the degradation environment and started to degrade the tested biomaterials. As shown in Figure 3B, the degradation ratio of BACG and BACG-B series increased rapidly compared to that of CMPHP in the initial 5 min. Then, the degradation slope of the four hemostatic materials gradually decreased in the next 20 min, indicating that the composite materials had a rapid degradation speed that gradually decreased over time. In addition, the degradation ratio of BACG and BACG-B series was over 90% at 25 min. However, as a positive control, commercial hemostat (CMPHP) had only a 76% degradation ratio at 25 min. Compared with the CMPHP, the prepared materials had better biodegradability. Taken together, the rapid degradation of the prepared materials met the requirement of hemostatic application in vivo.

### 3.2. Hemostatic Performance

#### 3.2.1. Whole Blood Coagulation In Vitro

Blood coagulation in vitro was characterized by observing blood flow after inversion of tubes that was incubated with hemostatic materials. All tested materials were graded as shown in Table 1 according to the ability to induce blood clotting [40].

**Figure 4:**
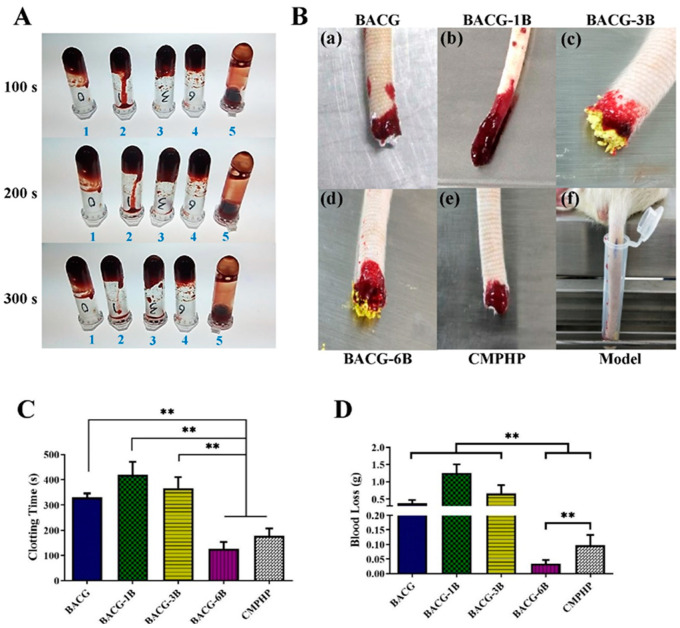
(**A**) Determination of clotting time in vitro. (#1–5) Compound microporous polysaccharide hemostatic powder (CMPHP), BACG-1B, BACG-3B, BACG-6B, blank control (BC) (*n* = 3); (**B**) photographs of hemostatic performance (**a**–**e**) BACG, BACG-1B, BACG-3B, BACG-6B, CMPHP, and (**f**) model of hemostasis in rats; (**C**) clotting time; (**D**) blood loss (mean ± SD, ** *p* < 0.01, * *p* < 0.05, *n* = 6).

As shown in Figure 4A, no clots formed within 600 s in tube #5, which was incubated without hemostatic materials as blank control, while the blood still kept flowability (Grade: Noneffective). Most blood could be stopped by BACG-1B within 100 s, but there was still mildly bleeding in 200 s. Finally, complete hemostasis was achieved at 300 s (Grade: Sufficient). The hemostatic block of BACG-3B showed rapid hemostasis in the first 100 s, but the clot was not strong; one side of the clot slowly slid down over time (Grade: Fairly good). Complete hemostasis was successfully achieved by BACG-6B within 100 s (Grade: Great). The blood clot was completely formed without any sign of downward movement, which indicated BACG-6B could stop bleeding immediately. Moreover, the blood incubated with CMPHP was mostly stopped at 100 s with slight bleeding (Grade: Fairly good), which proved BACG-6B had a strong ability to stop bleeding even compared to the commercial hemostasis. Higher berberine proportion led to shorter coagulation time, suggesting that berberine might form π–π hydrophobic interaction and replace Ca^2+^ hydrophilic interaction, which will benefit cell adhesion and coagulation. Therefore, these results demonstrated that berberine is a key factor (cross-linking regulator) in enhancing the hemostatic ability of BACG.

#### 3.2.2. Rat Tail Amputation Model

To further confirm the bleeding control ability of BACG-B, a rat tail amputation experiment was conducted to observe the hemostatic efficiency of biomaterials (BACG-1B, BACG-3B, BACG-6B, BACG, and CMPHP) in vivo. Figure 4B(f) shows the cut tails naturally hung down and the wound was gently touched with various hemostatic materials in the tube to achieve noncompressive hemostasis. As displayed in Figure 4B, the yellow powder of BACG-6B was comprehensively covered in the rat tail wound with less blood loss at the bleeding site, while large and unsolid blood clots were formed by BACG-1B and CMPHP, indicating the fast and excellent hemostatic performance of BACG-6B. 

The clotting time of test groups is shown in Figure 4C. BACG-6B and CMPHP displayed a vastly remarkable difference from the other three materials. As expected, the blank control (BC) could not stop bleeding over 10 min. The order of the average clotting time was as follows: BACG-6B (126.17 s) < CMPHP (178.17 s) < BACG (330.33 s) < BACG-3B (366.00 s) < BACG-1B (419.17 s) < BC (>600 s), which demonstrated that BACG-6B could obviously shorten the clotting time. Furthermore, the data of blood loss is presented in Figure 4D. The amount of blood loss of rats by using BACG-6B and CMPHP was significantly lower than that of other groups. Therefore, compared with the other materials we prepared, BACG-6B could observably shorten the clotting time and reduce the amount of bleeding. Moreover, the surface of BACG-6B, which contained numerous quaternary amine ions from berberine, could interact with negatively charged cell membranes, forming a “catalytic potential” polar framework, and could further lead to interface stimulation and activation of coagulation factors, which would benefit the hemostatic performance of BACG-6B.

#### 3.2.3. Platelet Aggregation and Adhesion Test

**Figure 5:**
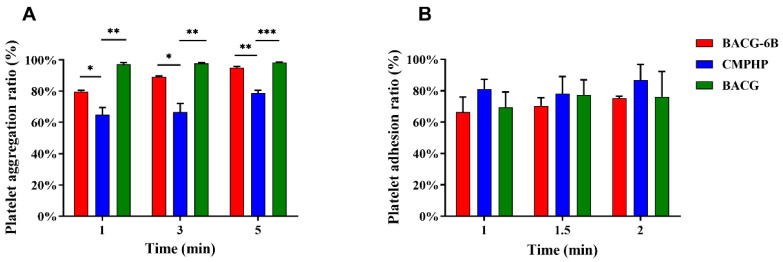
(**A**) Platelet aggregation test; (**B**) platelet perfusion adhesion test (mean ± SD, *** *p* < 0.001, ** *p* < 0.01, * *p* < 0.05, *n* = 3).

According to the above results, BACG-6B, BACG, and the positive control CMPHP were selected for a platelet aggregation and adhesion test. As illustrated in Figure 5A, all the materials caused platelet aggregation. Among them, BACG-6B showed a strong ability to aggregate platelets as its aggregation ratio increased to 95% in 5 min. However, CMPHP only showed a moderate ability to aggregate platelets (60%–77%). Compared with the previous two materials, BACG showed the highest aggregation ratio (>95%), with no significant change over all test periods. The rough surface of the two prepared hemostatic microspheres and their contained SCC/gelatin could help them to capture the platelets and further form platelet aggregation. 

Platelet adhesion is as important as platelet aggregation in the coagulation process [41]. As demonstrated in Figure 5B, the platelet adhesion ratio of BACG-6B was about 70% in 2 min. Besides, the overall adhesion trend of BACG was similar to that of BACG-6B. In addition, the adhesion ratio of CMPHP was a little higher than that of two prepared materials, but there was no statistical difference. In short, the significant platelet aggregation and adhesion on the surface of BACG and BACG-6B indicated the hydrophilic characteristic of the materials, which facilitated hemostasis and wound healing.

### 3.3. Antibacterial Activity

Berberine is the main antibacterial constituent of BACG-B, which has broad-spectrum antibacterial activity, especially against Gram-positive bacteria like *S. aureus* [42]. In this experiment, BACG-6B with a good hemostatic effect was selected for an antibacterial test based on the previous results and determined by the preincubation plate pouring method [43]. Among the test samples, berberine, ampicillin, and BACG-6B all showed obvious bacteriostatic circles, and the bacteriostatic circles were in the following order: 1 mg/mL berberine > 1 mg/mL Amp^+^ > 10 mg/mL BACG-6B > 1 mg/mL BACG-6B. This indicated that the antibacterial effect of BACG-6B mainly came from berberine with dose-dependence. The diameters of the bacteriostatic circles for *S. aureus* were measured and are displayed in Table 2. 

BC stands for blank control. *D*_1_ and *D*_2_ mean the diameters of the *S. aureus* bacteriostatic circles in two parallel tests, and d means the diameter of the filter paper, which is 6 mm. The width of the bacteriostatic circle (*W*) was calculated according to Equation (7):

(*W* = (*D* − *d*)/2 (7)

According to Table 2, the average bacteriostatic circles against *S. aureus* formed by 1 mg/mL Ampicillin, 1 mg/mL berberine, 1 mg/mL BACG-6B, and 10 mg/mL BACG-6B were 9.75 mm, 10.75 mm, 5.25 mm, and 8.50 mm, respectively, indicating that all test samples had obvious anti-*S. aureus* effects. Moreover, berberine performed as the main component to enhance the antibacterial activity of the BACG, which was consistent with our previous findings [34]. 

### 3.4. Biocompatibility

#### 3.4.1. Cytotoxicity Evaluation

**Figure 6:**
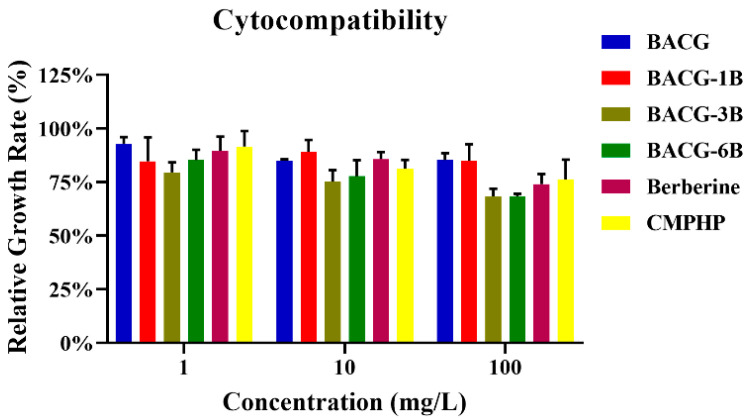
Cytocompatibility of hemostatic materials and drugs.

To evaluate the biocompatibility of the prepared hemostatic materials, MTT assays were performed for cytotoxicity testing. After the cells were exposed to the biomaterial or drug for 72 h, the cell density was in accordance with the cytocompatibility of the material. As displayed in Figure 6, BACG and BACG-1B showed no significant difference in cytotoxicity at different concentrations (*p* > 0.05). The cell’s viability of BACG-3B, BACG-6B, berberine, and CMPHP decreased with concentration increase. Surprisingly, the group with the lowest survival rate had a rate above 60% at the highest concentration (100 mg/L), and there was no significant difference between CMPHP and BACG-6B groups (*p* > 0.05), which indicated that all tested biomaterials could be used as safe hemostats.

#### 3.4.2. Hemolysis Test

**Figure 7:**
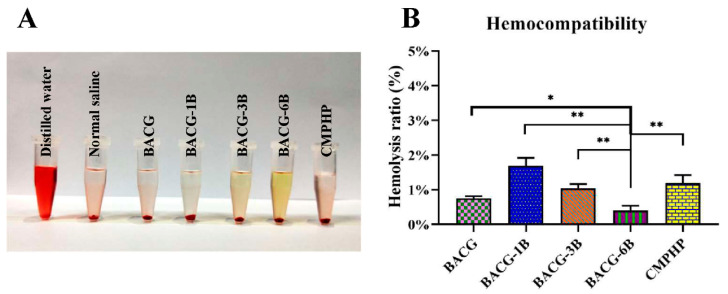
Hemocompatibility of hemostatic materials. (**A**) Photographs of hemolysis assays for test groups and control groups. (**B**) The hemolysis ratio of the corresponding biomaterials. (mean ± SD, ** *p* < 0.01, * *p* < 0.05, *n* = 3).

To further evaluate the hemocompatibility, a hemolysis test was conducted using 2% RES. As shown in Figure 7A, it was found that severe hemolysis was observed in the PC group while NC, BACG, BACG-1B, and CMPHP groups were almost colorless and transparent and BACG-3B and BACG-6B were slightly yellowish due to the dissolution of berberine. In Figure 7B, the calculation results showed that the hemolysis rates of all the test samples were below 5%. It demonstrated that composite materials did not cause hemolysis and had excellent hemocompatibility. In addition, the hemolysis ratio of BACG-6B was the lowest in all test groups and had significant difference even compared with CMPHP, indicating that BACG-6B had excellent biocompatibility in hemostatic application.

## 4. Conclusions

Biomimetic composite microspheres were prepared using alginate, carboxymethylcellulose, and gelatin as raw materials coated with berberine. The resulting hemostatic materials were characterized by SEM and FT-IR spectroscopy and exhibited rapid blood-sucking swelling and degradation. A series of platelet-related experiments in vitro indicated that our materials had the ability to enhance platelet adhesion and aggregation, especially BACG-6B. In particular, hemostasis analysis in vitro and in vivo further proved that BACG-6B could both significantly shorten the bleeding time and reduce the amount of bleeding. In addition, cytotoxicity evaluation and hemolysis test demonstrated that our materials exhibited excellent biocompatibility and safety. Most importantly, BACG-6B was also an ideal antibacterial agent to prevent infection after trauma. These results clearly indicate that BACG-6B is demonstrably the best due-functional biomaterial compared to other microspheres used in this work.

## Figures and Tables

**Table 1:** Coagulation performance grade.

Grade	Performance
Great	Complete hemostasis within 100 s
Fairly good	Almost complete hemostasis with mild bleeding within 100 s
Sufficient	Minor bleeding within 200 s and complete hemostasis within 300 s
Limited	No bleeding within 600 s
Noneffective	Bleeding over 600 s

**Table 2:** Bacteriostatic circles.

	1 mg/mL Ampicillin	1 mg/mL Berberine	1 mg/mL BACG-6B	10 mg/mL BACG-6B	BC
*D*_1_/mm	23.0	26.0	16.0	24.0	6.0
*D*_2_/mm	28.0	29.0	17.0	22.0	6.0

## Data Availability

The data presented in this study are available on request from the corresponding author.

## Supplementary Materials

The following are available online at https://www.mdpi.com/2073-4360/13/3/360/s1, Table S1: The proportion of hemolytic experimental materials.

## Abbreviations

SA	Sodium alginate
SCC	Sodium carboxymethyl cellulose
B	Berberine
BACG	Biomimetic alginate/carboxymethylcellulose/gelatin
BACG-B	Biomimetic alginate/carboxymethylcellulose/gelatin coated with Berberine
CMPHP	Compound microporous polysaccharide hemostatic powder
SEM	Scanning electron microscope
FT-IR	Fourier transform infrared
SR	Swelling ratio
DR	Degradation ratio
AGR	Aggregation ratio
ADR	Adhesion ratio
CV	Cell viability
HR	Hemolysis ratio
BA	Before aggregation
AA	After aggregation
BP	Before perfusion
AP	After perfusion
RBC	Red blood cell
RES	Rabbit erythrocyte suspension
PRP	Platelet-rich plasma
SD	Sprague Dawley
PBS	Phosphate-buffered saline
TS	Test sample
NC	Negative control
BC	Blank control
PC	Positive control
