# Supplementary material for: Berberine-Coated Biomimetic Composite Microspheres for Simultaneously Hemostatic and Antibacterial Performance
Source: Polymers (Basel). 2021 Jan 22;13(3):360. doi: 10.3390/polym13030360 (PMC7865669; doi:10.3390/polym13030360)
Supplement: Supplementary file 1 [file polymers-13-00360-s001.pdf]

**Supplemental Materials**

# Berberine-Coated Biomimetic Composite Microspheres for Simultaneously Hemostatic and Antibacterial Performance

**Xiaojian Zhang [1], Kaili Dai [1], Chenyu Liu [1], Haofeng Hu [2], Fulin Luo [2], Qifan Qi [2], Lei Wang [2,3], Fei Ye [2,3], Jia Jin [2,3,*], Jie Tang [1,*] and Fan Yang [1,*]**

1 Shanghai Engineering Research Center of Molecular Therapeutics and New Drug Development, East China Normal University, Shanghai 200062, China; 51184300155@stu.ecnu.edu.cn (X.Z.); 15988810463@163.com (K.D.); 51194300147@stu.ecnu.edu.cn (C.L.)

2 College of Life Sciences and Medicine, Zhejiang Sci-Tech University, Hangzhou 310018, China; 201920201014@mails.zstu.edu.cn (H.H.); 202020801049@mails.zstu.edu.cn (F.L.); 2018339901070@mails.zstu.edu.cn (Q.Q.); leiwang1986@hotmail.com (L.W.); yefei@zstu.edu.cn (F.Y.)

3 Zhejiang Provincial Key Laboratory of Silkworm Bioreactor and Biomedicine, Hangzhou 310018, China

* Correspondence: jjin@zstu.edu.cn (J.J.); jtang@chem.ecnu.edu.cn (J.T.); fyang@chem.ecnu.edu.cn (F.Y.)

**Supplemental Materials**

**Table S1**. The proportion of hemolytic experimental materials.

| | NC (0.9%NaCl) | PC ($ddH_2O$) | TS (test sample) | | | | |
|---|---|---|---|---|---|---|---|
| | | | BAG | BACG-1B | BACG-3B | BACG-6B | CMPHP |
| 2% RBCs/mL | 0.5 | 0.5 | 0.5 | 0.5 | 0.5 | 0.5 | 0.5 |
| 0.9%NaCl/mL | 0.5 | | 0.44 | 0.44 | 0.44 | 0.44 | 0.44 |
| Distilled water/mL | | 0.5 | | | | | |
| Test group/mL | | | 0.06 | 0.06 | 0.06 | 0.06 | 0.06 |

NC: negative control (0.9% NaCl solution); PC: positive control (distilled water).
